# Radiometal-Labeled Chitosan Microspheres as Transarterial Radioembolization Agents against Hepatocellular Carcinoma

**DOI:** 10.3390/gels8030180

**Published:** 2022-03-14

**Authors:** Hui-Wen Chan, Yi-Hsuan Lo, Deng-Yuan Chang, Jia-Je Li, Wen-Yi Chang, Chih-Hao Chen, Chih-Hsien Chang, Chuan-Lin Chen, Hsin-Ell Wang, Ren-Shyan Liu, Chun-Yi Wu

**Affiliations:** 1 Department of Biomedical Imaging and Radiological Sciences, National Yang Ming Chiao Tung University, Taipei Branch, Taipei 112, Taiwan; huiwen.be09@nycu.edu.tw (H.-W.C.); yihsuan.lo1992@gmail.com (Y.-H.L.); boxes129@gmail.com (D.-Y.C.); chchang@iner.gov.tw (C.-H.C.); clchen2@ym.edu.tw (C.-L.C.); hewang@ym.edu.tw (H.-E.W.); rsliu@vghtpe.gov.tw (R.-S.L.); 2 KeMyth Biotechnology Corporation, NHRI Incubation Center, National Health Research Institutes, Miaoli 350, Taiwan; uvpxyz@gmail.com; 3 Division of Infectious Diseases and Tropical Medicine, Department of Internal Medicine, Tri-Service General Hospital, National Defense Medical Center, Taipei 114, Taiwan; 4 Department of Nuclear Medicine, Taipei Veterans General Hospital, Taipei 112, Taiwan; nowkia@gmail.com (W.-Y.C.); npccjoe@gmail.com (C.-H.C.); 5 Institute of Nuclear Energy Research, Taoyuan 325, Taiwan

**Keywords:** transarterial radioembolization (TARE), hepatocellular carcinoma (HCC), radiolabeled chitosan microspheres (^111^In/^177^Lu-DTPA-CMS), theranostics

## Abstract

Transarterial radioembolization (TARE) is an emerging treatment for patients with unresectable hepatocellular carcinoma (HCC). This study successfully developed radiometal-labeled chitosan microspheres (^111^In/^177^Lu-DTPA-CMS) with a diameter of 36.5 ± 5.3 μm for TARE. The radiochemical yields of ^111^In/^177^Lu-DTPA-CMS were greater than 90% with high radiochemical purities (>98%). Most of the ^111^In/^177^Lu-DTPA-CMS were retained in the hepatoma and liver at 1 h after intraarterial (i.a.) administration. Except for liver accumulation, radioactivity in each normal organ was less than 1% of the injected radioactivity (%IA) at 72 h after injection. At 10 days after injection of ^177^Lu-DTPA-CMS (18.6 ± 1.3 MBq), the size of the hepatoma was significantly reduced by around 81%, while that of the rats in the control group continued to grow. This study demonstrated the effectiveness of ^177^Lu-DTPA-CMS in the treatment of N1-S1 hepatoma. ^111^In/^177^Lu-DTPA-CMS have the potential to be a superior theranostic pair for the treatment of clinical hepatoma.

## 1. Introduction

Hepatocellular carcinoma (HCC), the fourth leading cause of cancer mortality worldwide, is more common in East Asia, possibly due to the high prevalence of hepatitis B and C infections [1,2]. Among the treatment options, surgery is the first-line curative strategy; however, late-stage patients with multiple metastatic lesions are not candidates. The efficacy of chemotherapy or radiotherapy is sometimes unsatisfactory due to drug resistance and serious adverse effects. Transarterial radioembolization (TARE), also called selective internal radiation therapy (SIRT), has been considered an alternative for the curative treatment of HCC. TARE can locally deliver radiation to kill the tumor while reducing the damage to the healthy liver [3,4,5]. Two commercial ^90^Y-labeled embolization agents, SIR-Sphere^®^ and Therasphere^®^, have been approved by the Food and Drug Administration (FDA) for clinical use [6,7]. In preclinical studies, Chang et al. developed biodegradable poly(vinylsulfonic acid)/poly(D, L-lactideco-glycolide) (PLGA) loaded with doxorubicin and ^188^Re radionuclide to treat GP7TB rat liver epithelial tumor [8]. Recently, De La Vega et al. also prepared ^188^Re-loaded biodegradable microspheres made of poly(L-lactic acid) (L-PLA) and PEGylated polycaprolactone (PEG-PCL) for the treatment of N1-S1 rat hepatoma [9].

Chitosan, a natural biopolymer, is derived from the deacetylation of chitin and has been widely applied in the field of nanomedicine because of its high biocompatibility and low cytotoxicity [10,11]. Under acidic conditions, the amino groups of chitosan will be ionized, resulting in a positively charged polymer [12]. This characteristic is a driving force for chitosan to bind to the cell membrane. In addition, chitosan is a biodegradable material that can be degraded by lysozyme in the human body. Jeong et al. found that injected chitosan microspheres started to degrade 3 months after intramuscular administration and reached complete degradation after 5 months [13]. For application in TARE, Hwang et al. developed ^131^I-labeled chitosan microspheres and exhibited superior tumor inhibition in an orthotopic McA-RH7777-fLuc tumor-bearing rat model [14].

Lutetium-177 (^177^Lu), a well-known theranostic radionuclide, releases medium-energy β-particles accompanying two photons (113 and 208 keV) for diagnostic purposes and has a suitable half-life (6.7 d) to be applied in the field of cancer treatment [15,16,17]. Compared to ^90^Y, imaging the distribution of ^177^Lu in the living body is more straightforward. We can easily track ^177^Lu-labeled microspheres and determine the dosimetry with noninvasive single photon emission computed tomography (SPECT) imaging [18,19]. To our knowledge, to date, no studies have been reported on the application of ^177^Lu-labeled drugs in radioembolization for the treatment of hepatoma.

Although ^177^Lu-chelated chitosan microspheres can be directly used for pretreatment scans to exclude patients with severe lung shunt, any unnecessary β-dose should be avoided for patients to attain the goal of as low as reasonably achievable (ALARA). Additionally, the calculation of the treatment dose would be more complicated if the survey dose needed to be considered. Therefore, we aimed to develop radiometal chelated (^111^In and ^177^Lu) chitosan microspheres as a diagnostic/therapeutic pair and to investigate their biological behaviors in an orthotopic N1-S1 hepatoma-bearing rat model in this study.

## 2. Materials and Methods

### 2.1. Materials

Low-molecular-weight chitosan (50–190 kDa) and glycol chitosan were purchased from Sigma-Aldrich Co. (St. Louis, MO, USA). Span^®^ 80 was obtained from Alfa Aesar (Lancashire, UK). All other chemicals were purchased from Sigma-Aldrich Co. (St. Louis, MO, USA). The instant thin-layer chromatography plate was obtained from Agilent Technologies (Glostrup, Denmark). ^111^In-InCl_3_ and ^177^Lu-LuCl_3_ were purchased from the Institute of Nuclear Energy (Taoyuan, Taiwan) and ITG Isotope Technologies Garching GmbH (Garching, Germany), respectively. Cell culture medium was purchased from Thermo Fisher Scientific (Waltham, MA, USA). Fetal bovine serum was obtained from GE Healthcare—HyClone Laboratories Inc. (Logan, UT, USA). ITLC-SG plate was purchased from Agilent Technologies (Glostrup, Denmark). RIMADYL was purchased from Zoetis (Parsippany, NJ, USA). BAYTRIL was purchased from Bayer HealthCare (Leverkusen, Germany).

### 2.2. Preparation of Chitosan Microspheres (CMS)

**Figure 1:**
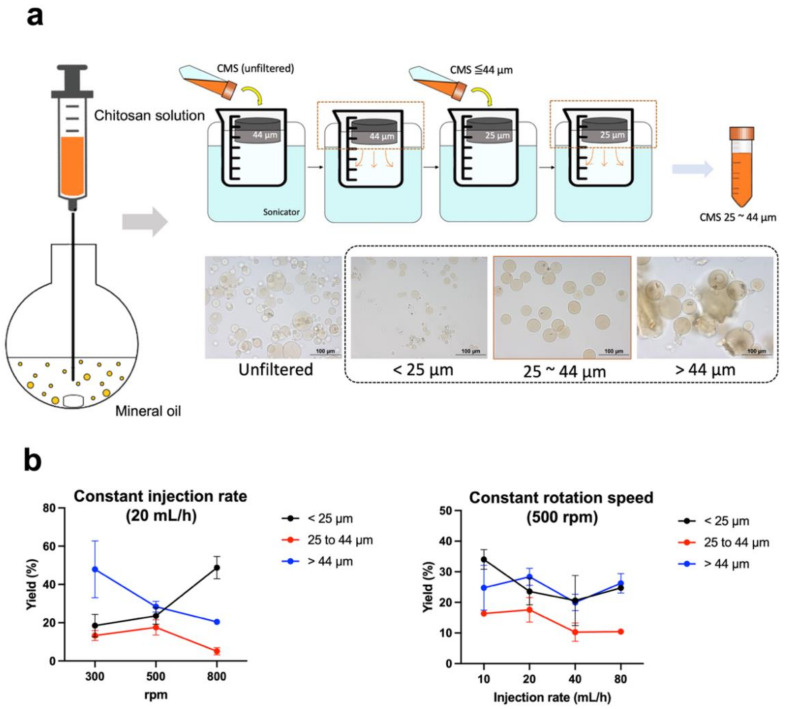
Preparation and physical characterization of chitosan microspheres. (**a**) Schematic diagram of the chitosan preparation process. After filtration by sieves, the microscopic images demonstrated that the CMS with inappropriate size have been excluded. Only the CMS having a size ranging from 25 to 44 µm were used in the following experiments. (**b**) Chemical yields of chitosan microspheres using different rotation speeds and injection rates. Unfiltered: crude product; <25 µm: smaller-sized CMS; >44 µm: larger-sized CMS.

CMS synthesis was based on the previously published water-in-oil emulsion method with some minor modifications (Figure 1a) [20,21]. Briefly, chitosan (90 mg) and 10 mg of glycol chitosan were dissolved in 5 mL of acetic acid solution (2%, *v*/*v*) and 0.5 mL of Tween^®^ 80. The chitosan solution was loaded into a flask containing 100 mL of mineral oil and 0.5 mL of Span^®^ 80 by a pump at a constant injection rate. After 30 min of stirring, 250 µL of glutaraldehyde was added and the reaction mixture was allowed to stir for another 105 min. The mixture was centrifuged at 760× *g* for 3 min. The collecting pellet was rinsed twice with isopropanol and ddH_2_O. The particles were sequentially passed through 44 µm and 25 µm sieves for size selection. CMS diameter was determined using an optical microscope (Olympus BX61) and scanning electron microscope (JEOL JSM-7600F). The CMS yield was calculated using the following equation:

$$\mathrm{Yield}=\frac{the\;wet\;weight\;of\;particles\;with\;sizes\;ranging\;25-44\;µm\;}{the\;wet\;weight\;of\;whole\;particles\;}\times 100\%$$

### 2.3. Preparation of DTPA-Modified CMS

p-SCN-Bn-DPTA dissolved in 0.05 M carbonate buffer (pH = 8.4, 0.5 mg/mL) was added to the CMS solution. The concentration of p-SCN-Bn-DPTA in CMS solution was around 100 mg/mL. The mixture was kept at 50 °C for 1 h. After the reaction, the solution was centrifuged at 3000× *g* for 3 min. The DTPA-CMS pellet was collected and rinsed with PBS three times to remove unbound chelates.

### 2.4. Radiolabeling of CMS

^111^In-InCl_3_/^177^Lu-LuCl_3_ and 100 mg of DTPA-CMS were added to 200 µL of citrate buffer solution (0.1 M, pH = 5.0). The reaction mixture was kept at 37 °C for 30 min. After the reaction, 20 µL of diethylenetriamine pentaacetic acid solution (DTPA, 10 mM) was added and the solution was kept at 37 °C for another 30 min to remove nonspecific-bound radiometals. The mixture was then centrifuged (3000× *g*) for 3 min. The pellet was rinsed twice with citrate buffer and then redissolved in normal saline to yield the final product. Radiolabeling efficiency was evaluated by radio thin layer chromatography (radioTLC) on the ITLC-SG plate with citrate buffer (0.1 M, pH = 4.5) as the mobile phase.

### 2.5. In Vitro Stability of ^111^In- and ^177^Lu-Labeled DTPA-CMS

The radiolabeled DTPA-CMS was incubated in either 4 °C normal saline or 37 °C FBS for 0, 1, 4, 8, 24, 48, 72, 120, and 168 h. At each time point, 0.3 mL of the incubated sample was aspirated and loaded into a 0.22 µm membrane filter, followed by a rinse with 1 mL of normal saline. The radioactivities of the filtrate and membrane filter were determined by a dose calibrator (CRC-25R, Capintec). The stability was calculated by the following equation:

$$\mathrm{Stability}\;\left(\%\right)=\frac{the\;radioactivity\;of\;filter}{the\;total\;radioactivity\;of\;filter\;and\;filtrate}\times 100\%$$

### 2.6. Cell Culture and Xenograft Inoculation

N1-S1 cells were cultured in Dulbecco’s Modified Eagle Medium (DMEM) supplemented with 10% fetal bovine serum (FBS), 10% fetal bovine serum, and 1% L-glutamine. Sprague-Dawley rats (SD rats, 250–300 g), receiving a subcutaneous injection of RIMADYL and BAYTRIL, were anesthetized with the inhalation of 2% isoflurane in O_2_ prior to the orthotopic inoculation of hepatoma. The liver was exposed from the hole created by a laparotomy. N1-S1 cells (2 × 10^6^) in 100 µL of serum-free DMEM were implanted in the left lobe of the liver. The T2-weighted (T2W) magnetic resonance imaging (MRI) was applied to non-invasively monitor the tumor growth on a scanner (Bruker 7T PET/MR, Billerica, MA, USA) at Taipei Veterans General Hospital, Taiwan. When the tumor diameter reached around 1 cm, the rats were selected for further experiments.

### 2.7. SPECT/CT Imaging of Radiolabeled CMS

SPECT/CT images were acquired using the imaging modality (Discovery NM/CT 670, GE) at Taipei Veterans General Hospital, Taiwan. To determine the in vivo stability of ^111^In-labeled CMS, static imaging of healthy SD rats (250–300 g) was performed at 1, 24, 48, and 72 h after the intravenous (i.v.) injection of 14.8 MBq of ^111^In-labeled CMS (^111^In-DTPA-CMS). A ^111^In-InCl_3_-containing vial (~1.11 MBq) was placed in a remote area of field of view (FOV) as an internal reference for quantification. The average signal intensity of pixels within the lung was quantified using RadiAnt Dicom Viewer (v.2020.2.3).

For monitoring the distribution of ^111^In-DTPA-CMS in N1-S1 hepatoma-bearing SD rats, static imaging was carried out at 1, 24, 48, and 72 h after intraarterial injection of 14.8 MBq of ^111^In-DTPA-CMS (30 mg). The procedure of intra-arterial injection was based on that of a previous study [9]. Briefly, rats inhaled 2% isoflurane (in O_2_) for anesthetization and were mounted in a supine position. A laparotomy was performed to expose the left lateral lobe of the liver. The gastroduodenal artery (GDA) was carefully separated from the portal vein (PV). The common hepatic artery (CHA) was ligated with a temporal vessel clamp. A PE-10 tubing, connected to a kit containing stirring radiolabeled chitosan microspheres, was inserted into GDA and fixed with a 2–0 suture. The suspension radiolabeled chitosan microspheres were slowly injected at a constant rate. After administration, the tubing and clamp on the CHA were removed and 3M Vetbond tissue adhesive was applied for the treatment of GDA wounds. The body wall and skin of the rats were closed with absorbable surgical sutures. The rats were immediately sacrificed after 72 h of imaging and the livers were excised for ex vivo imaging to determine the detailed distribution of activity.

### 2.8. Biodistribution of Radiolabeled CMS

Hepatoma-bearing rats were sacrificed at 1, 24, 48, and 72 h after the intraarterial injection of 5.6 MBq of ^111^In/^177^Lu-DTPA-CMS. Blood and organ samples were collected, cleaned, and weighted. Their radioactivities were measured by a gamma counter (Cobra II Auto-Gamma Counter, Perkin-Elmer Inc., Waltham, MA, USA). The uptake values were expressed as a percentage of the injected activity per gram of sample weight (%IA/g).

### 2.9. The Therapeutic Efficacy of ^177^Lu-DTPA-CMS

Hepatoma-bearing rats were randomly divided into two groups, which were treated with normal saline and 18.5 MBq of ^177^Lu-DTPA-CMS, respectively. The tumor burden was determined by animal MRI at days 0 and 10. The tumor inhibition effect in each group was calculated by the following equation:

$$\mathrm{Tumor}\;\mathrm{growth}\;\mathrm{inhibition}\;\left(\%\right)=\frac{V_{post}-V_{pre}}{V_{pre}}\times 100\%$$

where *V_pre_* and *V_post_* were the tumor volumes determined by MRI on days 0 and 10, respectively.

### 2.10. Hematoxylin and Eosin Staining

The hepatomas were excised from rats that received an i.a. injection of CMS (30 mg) at 72 h after administration and fixed with 10% formalin for paraffin embedding. The paraffin-embedded tissue sections were dewaxed at 70 °C, followed by deparaffinization and rehydration prior to staining with hematoxylin and eosin (H&E). The slide was viewed with a bright-field microscope (Olympus BX-61, Tokyo, Japan).

### 2.11. Dosimetry of ^177^Lu-DTPA-CMS

The uptake of tissue in biodistribution studies at each time point was used for the estimation of the absorbed dose of organs/tissues as well as the effective dose in a 73 kg adult using OLINDA/EXM 1.0 software. The sphere model in the software was applied to estimate the absorbed dose of hepatoma with various masses of spheroids.

### 2.12. Statistical Analysis

The Prisms (version 9.3.1) software was used for statistical analysis. All values were expressed as the mean ± standard deviation. A two-way ANOVA test was applied for the comparison between different groups. Differences with *p* < 0.05 were regarded as statistically different.

## 3. Results

### 3.1. Production and Radiolabeling of Chitosan Microspheres (CMS)

**Figure 2:**
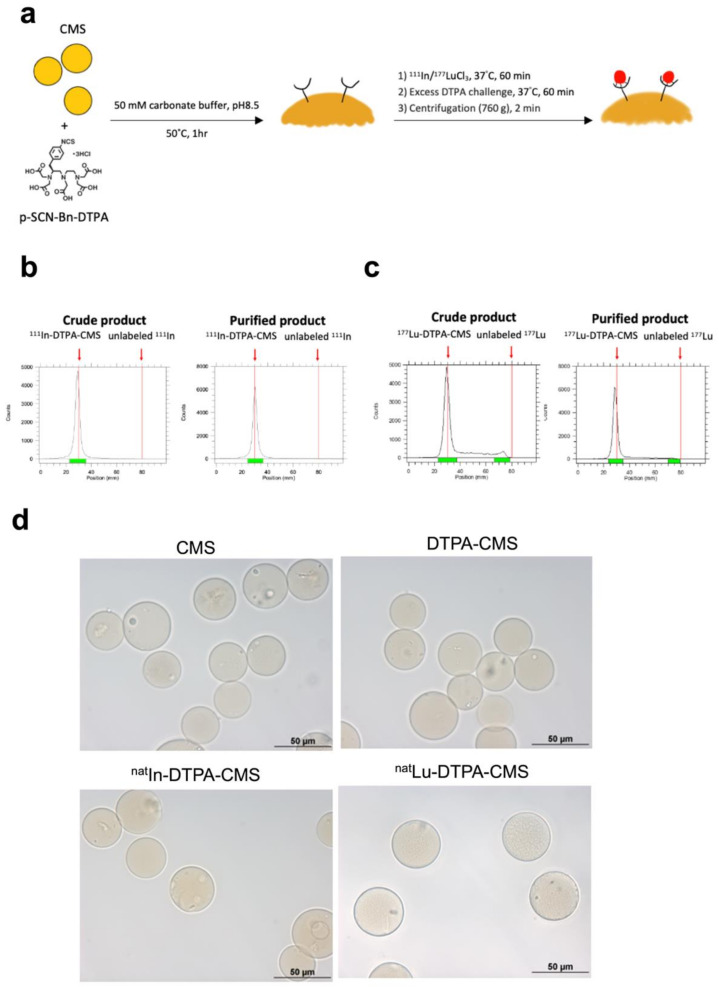
Preparation of radiolabeled chitosan microspheres. (**a**) The schematic diagram of radiolabeling. RadioTLC graphs of (**b**) ^111^In-DTPA-CMS and (**c**) ^177^Lu-DTPA-CMS before and after purification. Retention factor (Rf) values of ^111^In-DTPA-CMS, ^111^In-DTPA-CMS, unlabeled radioactive ^111^In, and unlabeled radioactive ^177^Lu are 0, 0, 1, and 1, respectively. (**d**) The size of unmodified chitosan microspheres (CMS), DTPA-modified chitosan microspheres (DTPA-CMS), and chitosan microspheres labeled with naturally existing indium (^nat^In-DTPA-CMS) and lutetium (^nat^Lu-DTPA-CMS). The images were obtained from a bright-field microscope.

We noticed that the stirring rate of 500 rpm resulted in the highest yield of CMS at any injection rate (Figure 1b). Regarding the fact that the elevated injection rate did not appear to improve the yield, we selected 20 mL/h as the injection rate for the preparation (Figure 1b). In the most optimal synthetic condition, the average diameter of the CMS was 36.5 ± 5.3 µm with a chemical yield of 17.6 ± 4.0 %. Figure 2a shows a schematic of the ^111^In/^177^Lu labeling procedure. The amount of DTPA on the CMS was around 3.4 × 10^18^ molecules/g CMS. The estimated surface modification efficiency was around 73%. The labeling efficiencies of ^111^In- and ^177^Lu-DTPA-CMS were greater than 90% (Figure 2b,c). After removal of the unbound radiometals and purification processes, the radiochemical purities of ^111^In- and ^177^Lu-DTPA-CMS were both over 98% (Figure 2b,c). The diameters of the CMS, DTPA-CMS, ^nat^In-DTPA-CMS, ^nat^Lu-DTPA-CMS, were 36.5 ± 5.3, 36.3 ± 5.2, 36.4 ± 4.2, and 38.5 ± 6.1, respectively (Figure 2d), indicating that the DTPA modification and radiolabeling did not affect the size of the CMS.

### 3.2. Stability of the Radiolabeled Chitosan Microspheres

**Figure 3:**
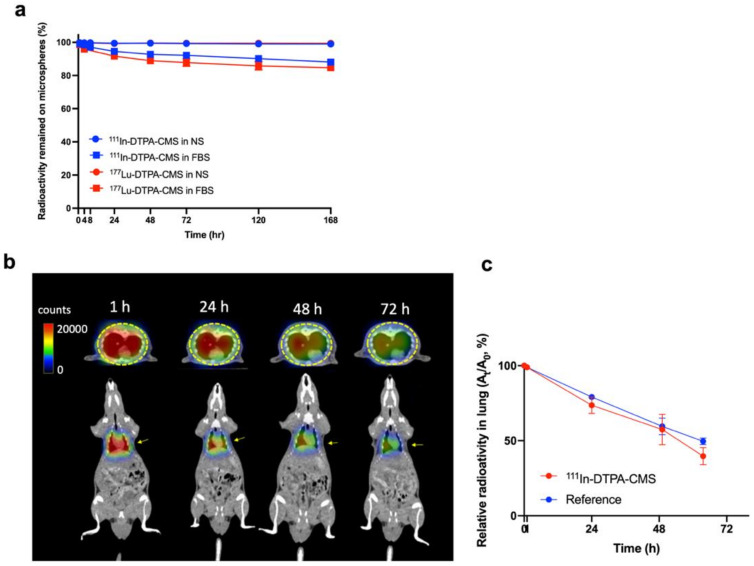
Stability of radiolabeled chitosan microspheres. (**a**) In vitro stability of ^111^In- and ^177^Lu-DTPA-CMS in either normal saline at room temperature or fetal bovine serum at 37 °C (*n* ≥ 5). (**b**) In vivo stability of radiolabeled chitosan microspheres determined by the SPECT images of rats receiving an intravenous injection of ^111^In-DTPA-CMS at 1, 24, 48, and 72 h post-injection (*n* ≥ 3). (**c**) Quantification of lung uptake at each time point. The reference values were obtained from the Eppendorf containing ^111^In-InCl_3_ placed in a remote area of the field of view.

The percentage of intact ^111^In-DTPA-CMS and ^177^Lu-DTPA-CMS were greater than 98% after a 168 h incubation in normal saline at r.t. but was relatively lower when incubated in FBS at 37 °C (Figure 3a). No differences were found between these two radiolabeled microspheres at all time points. However, the poor in vitro stability of the radiolabeled microspheres was observed when using DOTA as chelates (Figure S1). For in vivo stability, significant lung accumulation in healthy SD rats who received an i.v. injection of ^111^In-DTPA-CMS lasted for at least 72 h in SPECT imaging (Figure 3b), suggesting that the microspheres did not decompose in the body. Additionally, we quantified the radioactivity trapped in the lung and plotted a curve of radioactivity time (Figure 3c). The estimated effective half-life of ^111^In-DTPA-CMS, determined by the slope of the radioactivity–time curve, was 53.8 ± 3.8 h. The calculated biological half-life of ^111^In-DTPA-CMS was 284.7 ± 86.0 h, implying a superior in vivo stability of ^111^In-DTPA-CMS.

### 3.3. SPECT Imaging of Hepatoma-Bearing Rats

**Figure 4:**
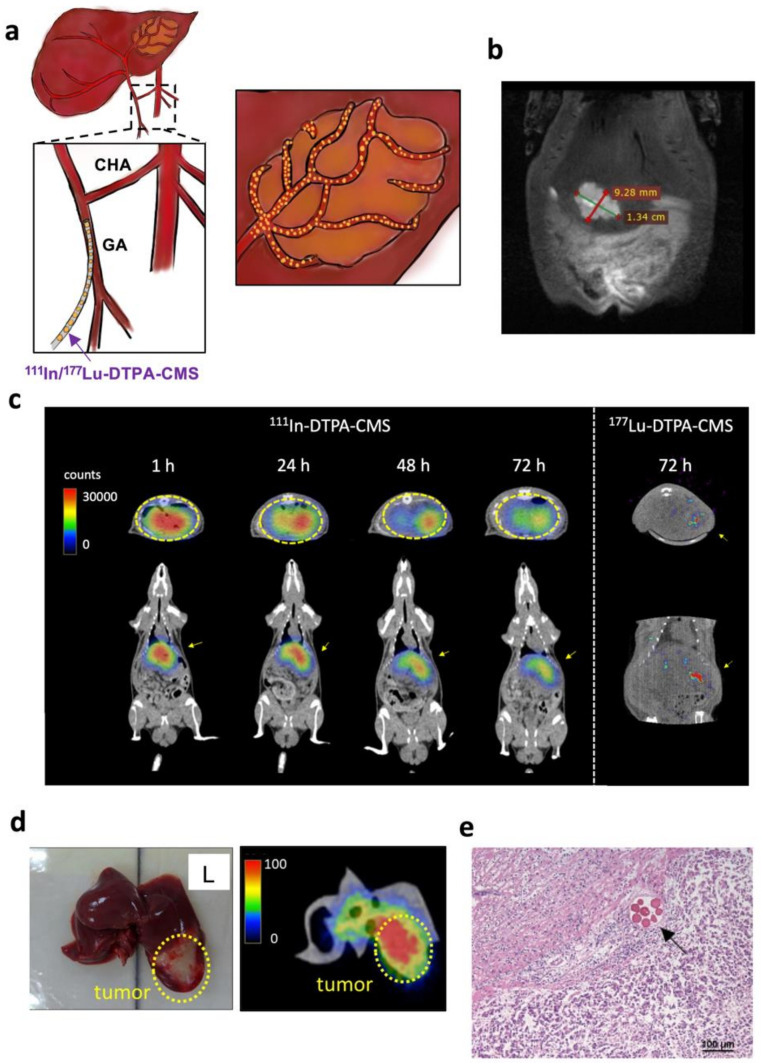
The distribution of ^111^In-DTPA-CMS in rats with N1-S1 hepatoma. (**a**) Schematic diagram of intraarterial injection of radiolabeled chitosan microspheres. (**b**) Representative T2-weighted MR image of rats with hepatoma on day 0. (**c**) SPECT/CT images of rats intraarterially injected with 14.8 MBq of ^111^In-DTPA-CMS at 1, 14, 48, and 72 h after injection (*n* = 3). MicroSPECT/CT images of rats intraarterially injected with 18.5 MBq of ^177^Lu-DTPA-CMS at 72 h after injection. (**d**) Ex vivo images of the liver injected with ^111^In-DTPA-CMS at 72 h post-injection. (**e**) H&E staining of the liver injected with ^111^In-DTPA-CMS. Microspheres were retained in the blood vessel of the tumor (arrow).

At 10 d after the inoculation of N1-S1 hepatoma cells, T2W MRI was performed to ensure hepatoma growth (Figure 4a,b). SPECT imaging showed the apparent radioactivity accumulated in the liver of rats with hepatomas at 1 h after intraarterial injection of ^111^In-DTPA-CMS and the microspheres remained firmly in place for at least 72 h (Figure 4c). No significant radioactivity retention was found in any other organ. After imaging, we immediately excised the liver and performed ex vivo imaging, demonstrating that most of the ^111^In-DTPA-CMS were retained in the hepatoma and only a few were detected within normal tissues (Figure 4d). H&E staining indicated that ^111^In-DTPA-CMS were selectively embolized into the blood vessels that supply blood to the tumor (Figure 4e). No significant vascular atrophy or fibrosis was observed in healthy tissues (Figure 4e). The estimated effective and biological half-lives of ^111^In-DTPA-CMS in hepatoma were 49.7 ± 0.6 and 190.6 ± 9.1 h, respectively.

### 3.4. Biodistribution Studies

The results obtained from the biodistribution studies were consistent with those observed in the SPECT imaging. Most of the ^111^In-DTPA-CMS and ^177^Lu-DTPA-CMS were retained in the hepatoma and liver (Table 1). ^111^In-CMS uptake in the tumor at 1 h and 72 h post-administration was 13.8 ± 6.3 and 12.7 ± 3.1 %IA/g, respectively, while that in the liver decreased slightly from 5.3 ± 2.1 to 3.6 ± 0.5 %IA/g. The tumor-to-liver ratio hit a plateau at 24 h after intraarterial injection. Radioactivity in blood was almost undetectable at each time point, resulting in a low uptake in other distant organs. Furthermore, the distribution and tumor uptake of ^177^Lu-DTPA-CMS 72 h after injection were similar to that of ^111^In-DTPA-CMS, indicating that ^111^In-DTPA-CMS are a good surrogate of ^177^Lu-labeled ones (Table 1). The liver shunt fractions (LSF) of ^111^In-DTPA-CMS and ^177^Lu-DTPA-CMS were ~1%, indicating there is only a small amount of these microspheres entering the lungs after intraarterial injection. 

### 3.5. The Therapeutic Efficacy of ^177^Lu-DTPA-CMS

**Figure 5:**
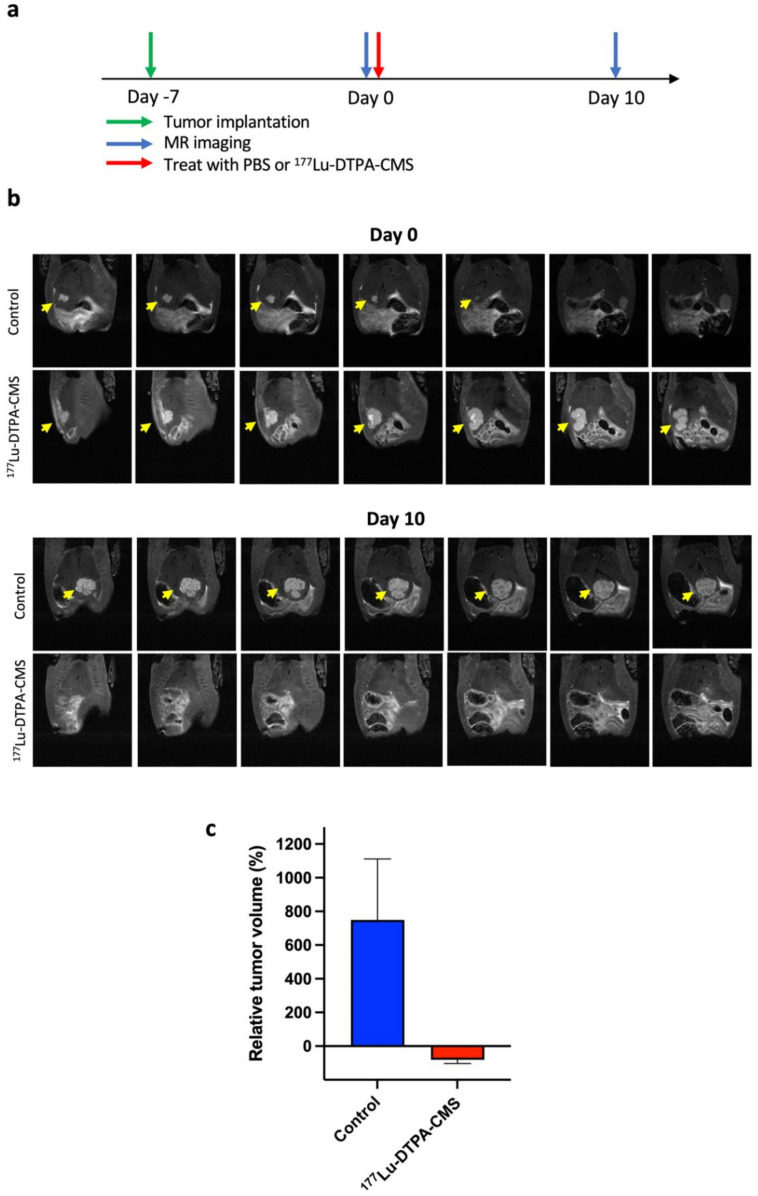
The therapeutic efficacy of ^177^Lu-DTPA-CMS against N1-S1 hepatoma. (**a**) The experimental scheme for the treatment. (**b**) T2-weighted MR imaging of rats treated with ^177^Lu-DTPA-CMS or normal saline; yellow arrows represent the tumor. (**c**) Quantification of changes in tumor size (*n* = 3).

To evaluate the therapeutic efficacy of ^177^Lu-DTPA-CMS in vivo, animal MRI was applied to non-invasively monitor the tumor size. The schedule for tumor implantation, ^177^Lu-DTPA-CMS administration, and imaging are illustrated in Figure 5a. Tumor shrinkage was found in the group treated with ^177^Lu-DTPA-CMS. At 10 d after the intraarterial injection of 30 mg of ^177^Lu-DTPA-CMS (18.6 ± 1.3 MBq), the size of the hepatoma was significantly reduced by approximately 81%, while that of rats in the control group increased by approximately 7 times (Figure 5b,c).

### 3.6. Estimation of Dosimetry in Human Organs in the Treatment of ^177^Lu-DTPA-CMS

The estimated dosimetry was derived from the results of the ^111^In-DTPA-CMS biodistribution studies and the values are summarized in Table 2. The liver received the highest absorbed dose of 2.34 mSv/MBq. The effective dose for a 73 kg adult is 0.14 mSv/MBq. The estimated dose for a 4 g tumor is 1.01 mGy/MBq (Table 2). As the tumor burden increases, the tumor absorbed dose decreases.

## 4. Discussion

With regard to the high prevalence of hepatocellular carcinoma in East Asia, an effective radioembolization agent that can eliminate inoperable hepatoma is warranted. Several radiolabeled biodegradable materials have been reported as radioembolization agents against hepatocellular carcinoma. For example, Chiang et al. applied the water-in-oil-in-water (W/O/W) emulsion method to provide poly(vinylsulfonic acid)/poly(lactide-coglycolide) microspheres loaded with ^188^Re and doxorubicin (^188^Re-Dox@PVSA/PLGA) and determined its therapeutic efficacy in rats with GP7TB hepatoma [8]. After i.a. injection, tumor accumulation of ^188^Re-Dox@PVSA/PLGA dropped from 12.1 %ID/g at 1 h p.i. to 7.6 %ID/g at 48 h p.i. After 4 weeks, a significantly delayed tumor growth was observed in the treated group. Emulsification is a relatively mature method for the preparation of chitosan microspheres [22,23]. However, this method would lead to certain disadvantages, such as somewhat complicated procedures, particle sizes in a wide range, and a limited drug-loading capacity. To our knowledge, the number of works using radiolabeled chitosan as TARE materials is limited. We believe the main reason is that the wide range of sizes of chitosan microspheres would greatly affect its biodistribution. The undesired distribution in the living body may cause healthy tissue damage. To solve this problem, we applied two sieves to exclude particles with inappropriate sizes after emulsion in the present study. Although filtration results in a relatively low chemical yield of the final product (~17%), we noticed that there was no unreasonable distribution, such as shunts, in the imaging or biodistribution studies (Figure 3, Figure 4 and Table 1). The size of ^nat^Lu-DTPA-CMS (38.5 ± 6.1 µm) is similar to the diameter of tumor blood vessels (30–40 µm), suggesting that these radiolabeled microspheres can be retained in the hepatoma as an embolization agent.

Amor-Coarasa et al. prepared DOTA-chelated chitosan microspheres using the water-in-oil (W/O) emulsion crosslink method for ^90^Y-labeling [24]. The intact percentage of ^90^Y-labeled chitosan microspheres (^90^Y-DOTA-CMS) in PBS after 72 h of incubation remained greater than 90% [24]. However, a marker reflecting the distribution of the “toxic” therapeutic agent is very important for clinical TARE. For example, ^99m^Tc-macroaggregated albumin (MAA) imaging is a routine procedure prior to ^90^Y-microspheres therapy because it can detect unfavorable extrahepatic distributions, which are sometimes caused by shunts and may lead to complications. In clinical settings, patients with high LSF (>20%) are not candidates for TARE since the radio-microspheres would be accumulated in the lung, causing severe radiation pneumonitis. Garin et al. indicated that the estimated dosimetry calculated based on ^99m^Tc-MAA SPECT/CT imaging can be applied to predict the overall survival of HCC patients who received glass-type ^90^Y-microsphere treatment [25]. However, the differences between ^99m^Tc-MAA and ^90^Y-microspheres in physical properties, including the size, density, and amount of injected particles, still affect the ability of ^99m^Tc-MAA imaging to predict the distribution of ^90^Y-microspheres, as well as prognosis. Haste et al. reported that ^99m^Tc-MAA SPECT/CT imaging would be a good predictor of normal liver parenchyma rather than hepatoma in HCC patients treated with glass ^90^Y-microspheres [26]. Compared to ^90^Y, lutetium-177 may be the most attractive radioisotope for theranostic applications, as it has moderate β-energy (0.497 MeV) for therapy and optimal γ rays (0.208 MeV) for imaging. A lot of ^177^Lu-labeled small molecules, peptides, or antibodies have been used for cancer therapy [15]. However, this is the first study to develop ^177^Lu-conjugated chitosan microspheres for TARE.

Considering that free radiometal in the living body would increase the dose to healthy tissues and could not reflect the real distribution of chitosan microspheres, we selected DTPA as the chelate for both ^111^In- and ^177^Lu-labeling instead of DOTA (Figure S1). The labeling efficiencies of ^111^In-DOTA-CMS were greater than 98% after a 30 min reaction at either 40 °C or 50 °C. We also noticed that the increased reaction temperature results in a higher in vitro stability of ^111^In-DOTA-CMS in FBS. The percentages of intact ^111^In-DOTA-CMS, prepared at 40 °C and 50 °C, decreased to 75% and 65% 72 h after incubation in FBS, respectively, and were not comparable with that of ^111^In-DTPA-CMS (94.3 ± 0.2%). A possible explanation is that the thermodynamic stability constant (Log_KML_) of the [^111^In(III)-DTPA] complex (29.0) was significantly higher than that of the [^111^In(III)-DOTA] one. In addition, the logarithmic KML of [^177^Lu(III)-DTPA] is similar to that of [^177^Lu(III)-DOTA], suggesting that DTPA is an optimal chelate for radiolabeling ^111^In and ^177^Lu. We found that the stability of ^177^Lu-DTPA-CMS was almost identical to that of ^177^Lu-DOTA-CMS (Figure 3). We also performed an orthotopic hepatoma treatment using an intraarterial injection to precisely deliver ^177^Lu-DTPA-CMS to the hepatoma. Regarding that the average range of β-rays is 670 μm [27], ^177^Lu-based TARE can be regarded as ideal brachytherapy with limited damage to nearby tissues compared to ^90^Y. The estimated liver absorbed doses for ^90^Y- and ^188^Re-CMS were 14.3 and 12.1 mSv/MBq (data not shown), respectively, which are significantly higher than that of ^177^Lu-DTPA-CMS. The effective half-life of ^111^In-DTPA-CMS in the lung (i.v. injection) and liver (i.a. injection), derived from SPECT imaging, was around 54 and 50 h, respectively, which were close to the physical half-life of ^111^In (67.3 h), implying that their superior in vivo stabilities lead to long-term retention. ^111^In-DTPA-CMS was found to have a shorter effective half-life in the liver than in the lung. Higher metabolic activity and blood flow in the liver may explain this phenomenon. Moreover, in the present study, the LSFs of ^111^In- and ^177^Lu-DTPA-CMS were approximately 1% (Table 1). The dosimetry studies also indicated the radiation dose of the lung was similar to that of other distant normal organs (Table 2), showing the particle size was suitable to use in HCC radioembolization without severe lung damage. SPECT/CT imaging and biodistribution studies showed a similar pattern in the tumor accumulation between ^111^In-DTPA-CMS and ^177^Lu-DTPA-CMS (Figure 4c and Table 1) and demonstrated that ^111^In-DTPA-CMS could be a reliable surrogate of ^177^Lu-DTPA-CMS. Compared to ^99m^Tc-MAA/^90^Y-microspheres, our theranostic pair shares identical material; thus, issues of different size, shape, and density between the diagnostic/prediction agent and therapeutic regimen no longer exist.

This study still has several limitations. First, unlike the standard clinical procedure, we manually injected ^177^Lu-DTPA-CMS into the gastroduodenal artery of hepatoma-bearing rats using a syringe because it is difficult to use angiography in the animal model. Therefore, we cannot precisely control the distribution of ^177^Lu-DTPA-CMS in the normal liver, causing an overestimation of the liver dose. Second, we did not determine the therapeutic efficacy of ^177^Lu-DTPA-CMS against multiple hepatoma lesions in the present study due to the difficulties of tumor inoculation. A further study is warranted to investigate this issue. Lastly, the selected treatment dose is based on the human dose and is tailored by body surface area calculation. We did not perform the maximal tolerance dose studies to find the optimal dose in this proof-of-concept study.

## 5. Conclusions

In this study, we applied a simple filtration to produce uniform-sized chitosan microspheres. We also prepared ^111^In- and ^177^Lu-DTPA-CMS with acceptable radiochemical yields. Current data demonstrated that ^111^In-DTPA-CMS is a reliable surrogate for pre-treatment evaluation. The intraarterial injection of ^177^Lu-DTPA-CMS into an orthotopic hepatoma-bearing rat model exhibited long retention and superior therapeutic efficacy. There were no severe side effects on normal tissues. The findings of this study suggest that ^177^Lu-DTPA-CMS could be a novel radioembolization agent against hepatoma and ^111^In- and ^177^Lu-DTPA-CMS could be a promising theranostic pair for clinical use.

## Figures and Tables

**Table 1:** Radioactivity distribution of ^111^In- and ^177^Lu-DTPA-CMS in rats with hepatoma N1-S1 after intraarterial injection.

	^111^In-DTPA-CMS ^a^				^177^Lu-DTPA-CMS
Organ ^b^	1 h	24 h	48 h	72 h	72 h
Blood	0.01 ± 0.01	0.01 ± 0.00	0.01 ± 0.00	0.01 ± 0.00	N.D. ^c^
Heart	0.02 ± 0.02	0.01 ± 0.01	N.D.	N.D.	N.D.
Lung	0.34 ± 0.15	0.33 ± 0.31	0.11 ± 0.01	0.15 ± 0.20	0.04 ± 0.04
Liver	5.31 ± 2.07	3.10 ± 0.52	3.15 ± 0.75	3.57 ± 0.51	4.48 ± 0.94
Stomach	0.66 ± 0.82	0.02 ± 0.03	0.17 ± 0.23	0.24 ± 0.28	0.25 ± 0.50
S.I.	1.08 ± 1.67	0.01 ± 0.01	0.03 ± 0.06	0.01 ± 0.00	0.04 ± 0.07
L.I.	0.41 ± 0.79	0.01 ± 0.01	0.01 ± 0.01	0.01 ± 0.00	0.00 ± 0.01
Spleen	1.13 ± 2.25	0.01 ± 0.00	0.05 ± 0.02	0.03 ± 0.03	0.09 ± 0.16
Pancreas	1.40 ± 2.69	0.01 ± 0.01	0.14 ± 0.15	0.09 ± 0.16	0.26 ± 0.52
Kidney	0.29 ± 0.51	0.05 ± 0.00	0.06 ± 0.02	0.09 ± 0.03	0.05 ± 0.09
Bladder	0.11 ± 0.20	0.05 ± 0.08	0.00 ± 0.01	0.01 ± 0.00	N.D.
Muscle	0.02 ± 0.04	N.D.	N.D.	N.D.	N.D.
Bone	0.07 ± 0.14	0.01 ± 0.01	0.00 ± 0.00	0.01 ± 0.00	0.05 ± 0.09
Tumor	13.75 ± 6.31	12.71 ± 6.37	12.87 ± 3.80	12.65 ± 3.13	13.11 ± 6.53
T/L ratio	2.58 ± 0.83	4.09 ± 2.07	4.06 ± 0.77	3.71 ± 1.53	2.91 ± 1.32
LSF(%) ^d^	0.83 ± 0.82	0.75 ± 0.26	0.79 ± 0.24	1.01 ± 1.08	0.05 ± 0.05

^a^ The values are expressed as mean ± S.D. (*n* = 4) and the unit was the percentage of injection activity per gram of organ (%IA/g). ^b^ S.I.: Small intestine; L.I.: large intestine; T/L ratio: tumor-to-liver ratio. ^c^ N.D.: Not detected. ^d^ Lung shunt fraction (%) = $\frac{A_{lung}}{A_{lung}+A_{liver}}\times 100\%$ (A is the radioactivity in organs).

**Table 2:** Radiation dose estimates for intra-arterial injection of ^177^Lu-DTPA-CMS in adult humans ^a^.

Organs ^b^	Dose (mSv/MBq)
Adrenals	0.04
Brain	0.01
Breasts	0.02
Gallbladder wall	0.06
LLI wall	0.01
Small intestine	0.02
Stomach wall	0.02
ULI wall	0.02
Heart wall	0.03
Kidneys	0.03
Liver	2.34
Lungs	0.09
Muscle	0.01
Ovaries	0.01
Pancreas	0.11
Red marrow	0.01
Osteogenic cells	0.04
Skin	0.01
Spleen	0.01
Testes	0.01
Thymus	0.02
Thyroid	0.01
Urinary bladder wall	0.01
Uterus	0.01
Tumor (4.0 g) ^c^	1.01
Total body	0.08
Effective dose	0.14

^a^ Radiation dosimetry was converted from ^111^In-DTPA-CMS biodistribution in a 0.3 kg rat to 73 kg male adults. ^b^ LLI: Lower large intestine; ULI: upper large intestine. ^c^ The dose absorbed by the tumor was obtained using the sphere model. The unit is mGy/MBq because no organ weighting factor is available for the sphere model.

## Data Availability

Not applicable.

## Supplementary Materials

The following supporting information can be downloaded at: https://www.mdpi.com/article/10.3390/gels8030180/s1. Figure S1: In vitro stabilities of ^111^In-DOTA-CMS prepared by various conditions in normal saline (NS) or fetal bovine serum (FBS). Figure S2: T2-weighted MR imaging of rats treated with normal saline (*n* = 3). Yellow arrows represent tumor lesions. Figure S3: T2-weighted MR imaging of rats treated with ^177^Lu-DTPA-CMS (*n* = 3). Yellow arrows represent tumor lesions.
