# Supplementary material for: Radiometal-Labeled Chitosan Microspheres as Transarterial Radioembolization Agents against Hepatocellular Carcinoma
Source: Gels. 2022 Mar 14;8(3):180. doi: 10.3390/gels8030180 (PMC8953182; doi:10.3390/gels8030180)
Supplement: Supplementary file 1 [file gels-08-00180-s001.zip › gels-1613922-supplementary.pdf]

Supplementary Information

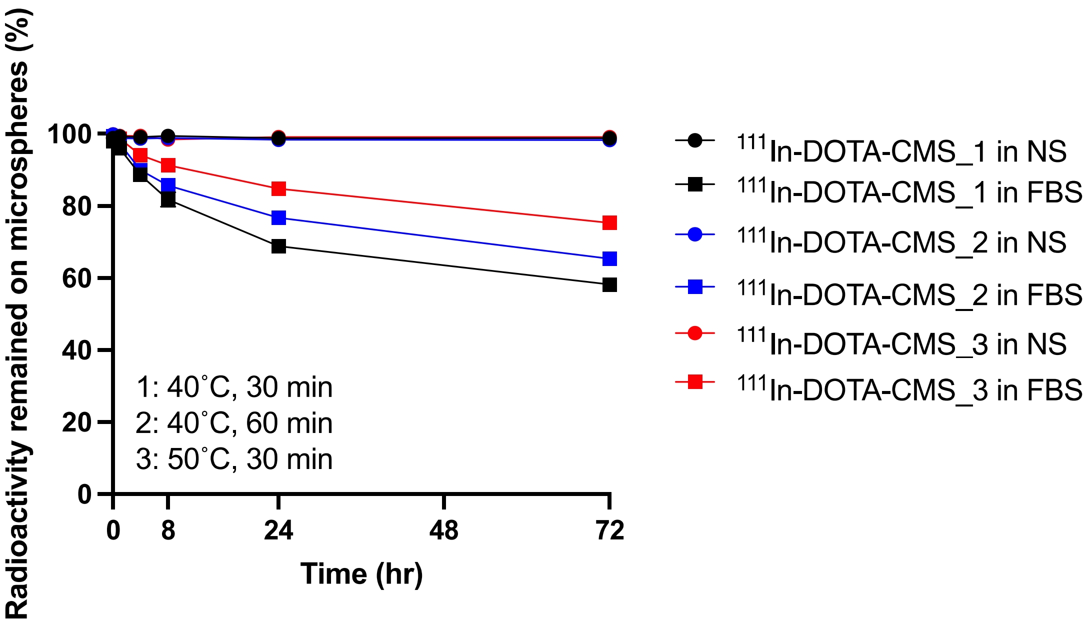

Figure S1. In vitro stabilities of ^{111}In-DOTA-CMS prepared by various conditions in normal saline (NS) or fetal bovine serum (FBS).

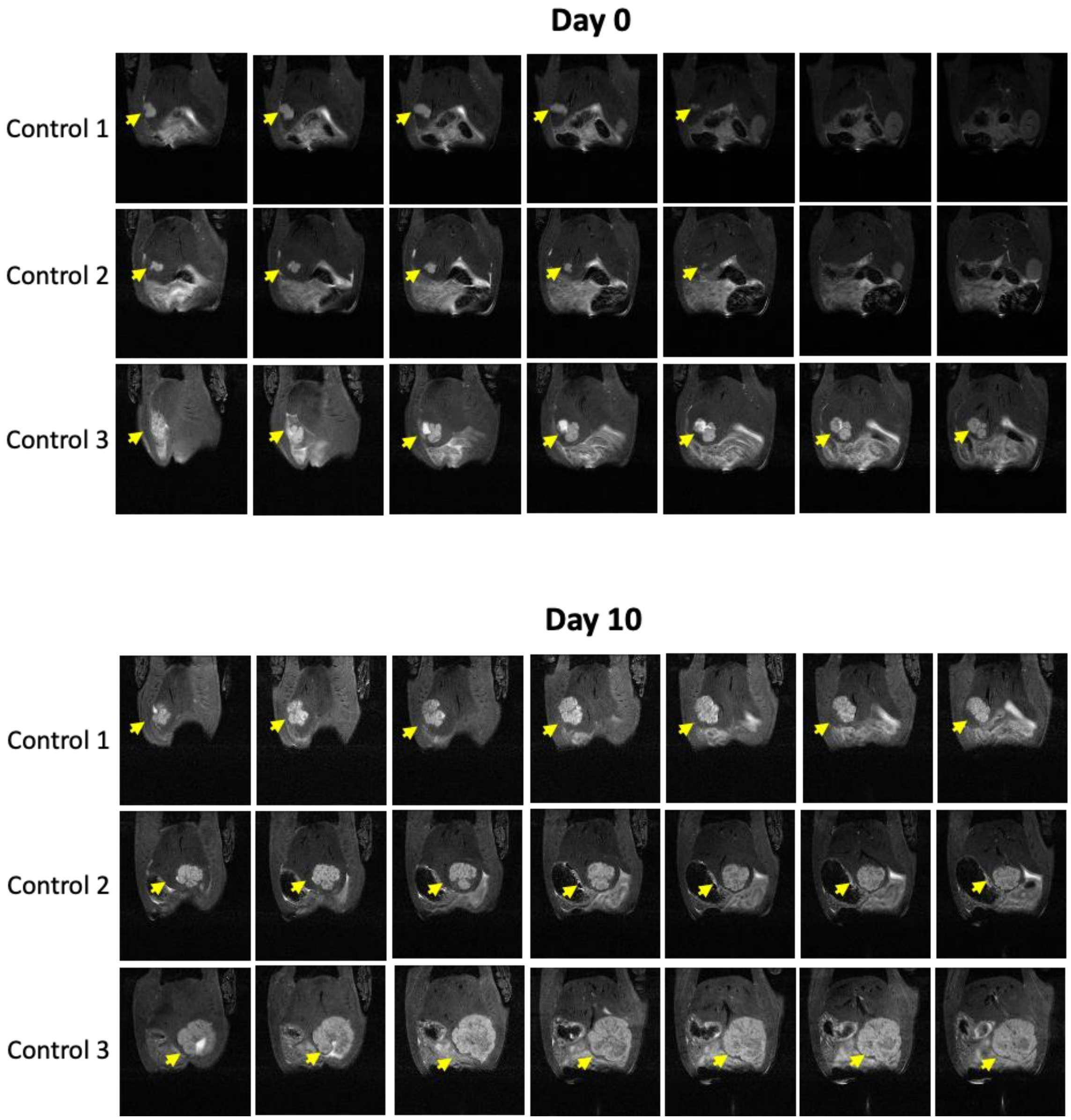

Figure S2. T2-weighted MR imaging of rats treated with normal saline (n=3). Yellow arrows represent tumor lesions.

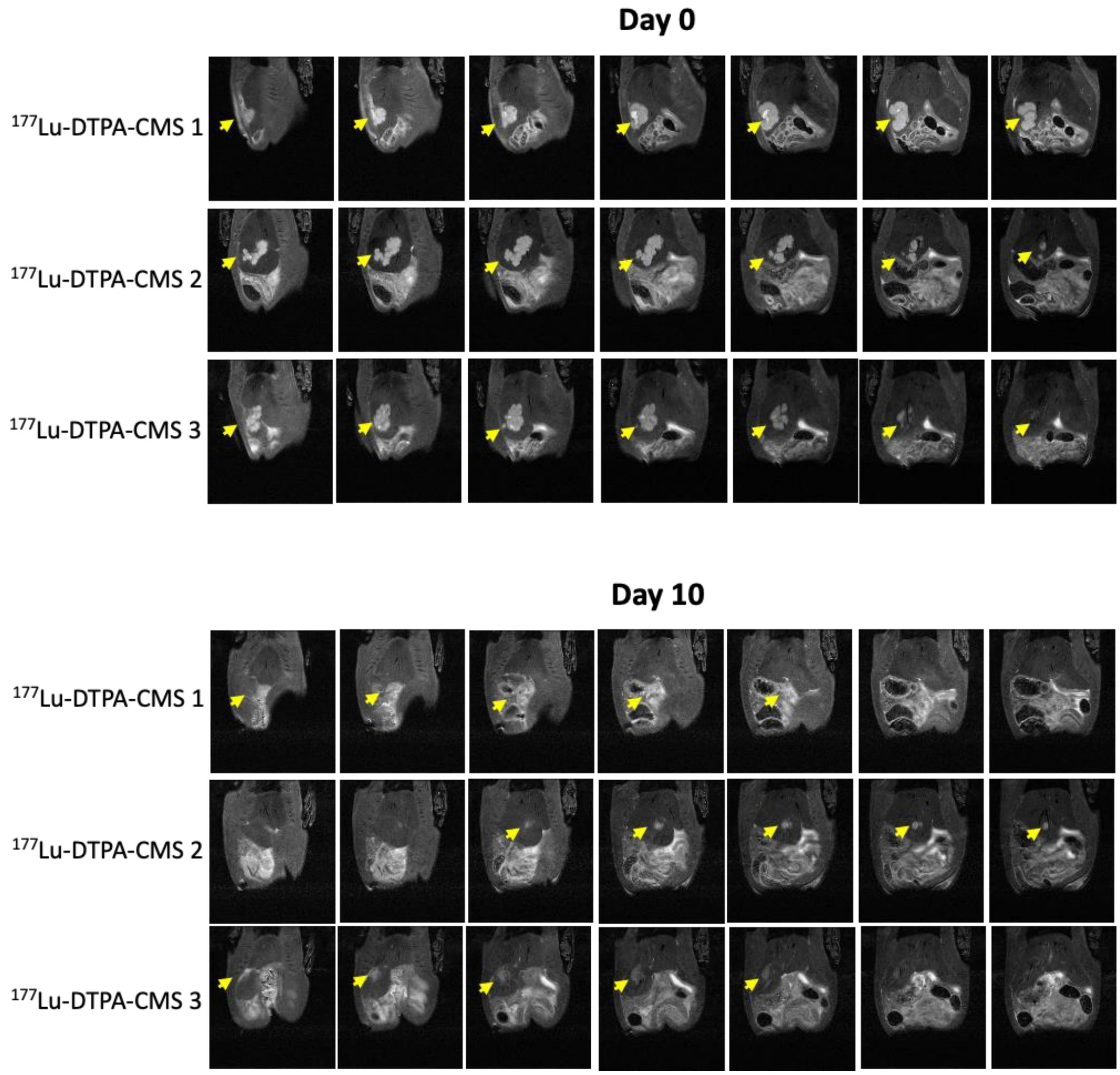

Figure S3. T2-weighted MR imaging of rats treated with ^{177}Lu-DTPA-CMS (n=3). Yellow arrows represent tumor lesions.
